# Impact of class cancellations on parents’ and children’ adaptation following an outbreak of the Omicron variant during the COVID-19 pandemic in Taiwan in April 2022

**DOI:** 10.1186/s12889-024-18976-y

**Published:** 2024-07-16

**Authors:** Kuo-Yu Chao, Tung-Yuan Hsiao, Sum-Fu Chiang, Wei Cheng

**Affiliations:** 1 Department of Nursing, Chang Gung University of Science and Technology, Taoyuan, Taiwan; 2 Division of Colon and Rectal Surgery, Chang Gung Memorial Hospital, Linkou, Taiwan; 3 National Alliance of Parents Organization, Taipei, Taiwan; 4 Graduate Institute of Clinical Medical Sciences, College of Medicine, Chang Gung University, Taoyuan, Taiwan; 5 School of Traditional Chinese Medicine, Chang Gung University, Taoyuan, Taiwan; 6 Department of Pathology, Kee-Lung Hospital, Ministry of Health and Welfare, 268 Shin-Erh Road, Keelung, 201 Taiwan; 7 School of Nursing, National Taipei University of Nursing and Health Sciences, Taipei, Taiwan; 8 Department of Nursing, Deh Yu College of Nursing and Health, Kee-Lung, Taiwan

**Keywords:** Parents, Children, Socioeconomic status (SES), Class cancellations, COVID-19

## Abstract

**Objective:**

To explore the impact on Taiwanese parents and children following an outbreak of the Omicron variant during the COVID-19 pandemic.

**Methods:**

Data were collected following class cancellations mandated by the Ministry of Education due to an outbreak of the Omicron variant of COVID-19 in April 2022. A national parent organization developed self-report survey questionnaire, “Impact of the Pandemic-related School Closures/Class Cancellations” (IPRSCCC), assessed parents’ perceived impact of school cancellations on their child/children’ and on their adaptation. The online survey was available between May 4 and May 9, 2022, in 20 districts throughout Taiwan.

**Results:**

A total of 2126 parents representing 2592 children responded. Total scores on the IPRSCCC were significantly higher for parents of children whose classes were cancelled (*n* = 891) compared with parents whose children continued in-person classes (*n* = 1053). Parents perceived the class cancellations of the child/children disrupted daily routine, learning loss and impacted academic motivation. They also reported emotional stress and no time for rest, which were associated with parental burnout. However for these parents, there were no significant differences in scores between parents living in low and high socioeconomic areas. Only the subscale score for disrupted daily routine was significantly higher for fathers, and emotional stress was significantly higher for parents with two, or ≥ 3 children. When academic impacts were examined using national examination scores for 12th grade students, the percent of students with scores of ≤ 6 in English, Chinese, and mathematics was higher in 2022 than in 2020.

**Conclusions:**

Higher IPRSCCC scores for parents of children whose classes were cancelled provides additional evidence of the impact of disruptions of in-person classes due to the COVID-19 pandemic. Examination scores confirmed class cancellations impacted academic performance.

**Supplementary Information:**

The online version contains supplementary material available at 10.1186/s12889-024-18976-y.

## Introduction

The novel coronavirus disease 2019 (COVID-19) originated in Wuhan, China in December 2019 and was declared a pandemic by the World Health Organization (WHO) on March 12, 2020 [1]. Many countries imposed regional or nationwide school closures to reduce the spread of the virus, named SARS-CoV-2, which causes severe acute respiratory syndrome (SARS) [2]. Taiwan has been one of the world's few countries that successfully combated the spread of COVID-19, having kept the total number of cases to the hundreds, rather than the thousands. In August 2020, the total deaths reported for the population of Taiwan was only 0.3 deaths per million [3].

However, the success of preventing the spread of the virus changed on April 20, 2021 when an outbreak of the alpha variant of the virus occurred and by May 17, 2021, there were more than 900 cases per week. A zero-tolerance response was in place during the 2021 outbreak, which cancelled all in-person attendance of classes once two confirmed cases of COVID-19 were reported for a student or faculty member, and the Ministry of Education (MOE) announced the closing of all schools (from preschools to universities) in Taiwan on May 18, 2021 [4], and schools remained closed until September 1, 2021, when COVID-19 was brought under control.

In March 2022, an outbreak of the Omicron variant of COVID-19 caused another rise in infections. However, by this time, the Taiwanese government had abandoned their policy of “zero-tolerance” for the virus and attempted a strategy that would allow for coexistence with the virus. This strategy applied more relaxed guidelines. If one student tested positive for COVID-19, their entire class was considered to be “close contacts” and students underwent home quarantine and classes were cancelled for 10 days until all students tested negative. Unfortunately, on April 28, 2022, Taiwan reported over 10,000 cases in a day for the first time since the pandemic began and in-person classes were partially or totally cancelled at 412 schools in 18 cities and counties [5]. Though the MOE said it would respect local governments and schools that chose to adjust the policy, parents had no choice to have their children attend school in-person or remotely. The reason was that most of schools cancelled the in-person classes if there were cases or outbreaks. In addition, by April 20, 2022 Taiwan had approved the Moderna vaccine for children aged 6–11 [6].

Parents globally have been profoundly impacted by school closures and class cancellations resulting from the pandemic due to the lack of external sources of support and the additional childcare responsibilities generated by school closures and class cancellations [2]. Children’s physical and mental health have also been shown to be negatively affected by school closures and class cancellations [7], with the most significant impact on younger students, those with learning disabilities, and those in lower socioeconomic groups [8]. During the pandemic, parents of children who were receiving virtual or combined instruction expressed a higher frequency of reporting a decline in their child's mental or emotional well-being [9], particularly among high school students [10]. A Systematic Review study by Hammerstein et al. [11] also revealed a detrimental impact of school closures on student achievement, particularly among younger students and those from low socioeconomic backgrounds.

The epidemic broke out in Taiwan later than most countries in the world because the government controlled it well. Therefore, during the school closure period in Taiwan, there have been studies to understand the impact of families on stress coping strategies and adaptation in the face of school closures caused by the epidemic. Coping strategies are divided into emotion-focused and problem-focused, then adaptation results could be achieved [12]. Therefore, we searched journals to gain a deeper understanding of this phenomenon, and developed a questionnaire suitable for Taiwan.

Open access data and longitudinal follow ups are vital to accumulate sufficient evidence on the impact of school closures and class cancellations on the health and well-being of children, adolescents, and their parents. Therefore, the purpose of this study was using designed questionnaire to examine parents’ perceived impact of class cancellations on their children’s adaptation to learning, and themselves’ adaptation to social or mental stress, following the outbreak of the Omicron variant of COVID-19 in April 2022 in Taiwan. Further, we would like to know if there were differences between fathers and mothers, parents with different socioeconomic status (SES), or parents with different number of children. Online subjective survey data were collected in May 2022. In addition, to examine if there were objective impacts on academic performance, 2020 and 2022 data from Taiwan’s national examinations of 9th grade and 12th grade students were collected.

## Methods

### The survey

**Fig. 1:**
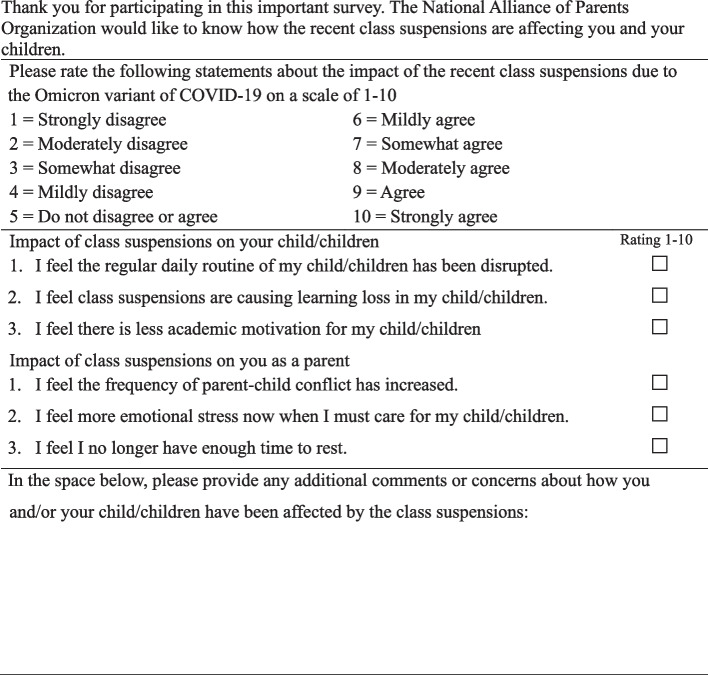
Contents of the Impact of Pandemic-Related School Closures/Class Cancellations (IPRSCCC) online survey

The National Alliance of Parents Organization (NAPO), a professional organization of parents, developed a self-report survey instrument intended to assess parents’ perceptions of the impact of sudden school closures and cancellations due to COVID-19 on their children and themselves. The 6-item survey, entitled “Impact of the Pandemic-related School Closures/Class Cancellations” (IPRSCCC), was comprised of statements regarding a parent’s perceived impact of school cancellations on their child/children (3 items: disrupted daily routine, learning loss and academic motivation) and on themselves (3 items: parent–child conflict, emotional stress, and no time for rest), which were mentioned in references [13–15]. Items were scored on a 10-point Likert scale from 1 = strongly disagree to 10 = strongly agree. Total scores ranged from 6 to 60, with higher scores indicating a greater impact of school closures on the family. The content validity index (CVI) of the survey items was examined by a panel of five experts, four with school-age children: two were experts with a PhD in education, and three with master’s degrees. The CVI was 0.89, indicating excellent validity of the items. Cronbach’s alpha for internal consistency for the child and parent categories was 0.863 and 0.792, respectively; the Cronbach’s alpha for the total IPRSCCC score was 0.846. Confirmatory factor analysis resulted in factor loadings for all six items from 0.688 to 0.887 (Supplementary table S1). Fit indices (FI) were 0.985 for goodness of fit index (GFI), 0.961 for adjusted GFI, normed and non-normed FI were both > 0.9. The overall fit indices of IPRSCC were in Supplementary table S2. Therefore, the survey had good reliability and validity and could serve as a tool for collecting data about parents’ perceptions about school closures. A second section of the IPRSCCC survey allowed parents to provide qualitative feedback about their personal experiences with the school cancellations. This was an open-ended question, asking parents if they had anything they would like to share about the impact of class cancellations on them as parents, their children, and their families. For details please see Fig. 1.

We measured parent report only, because parents worldwide have been profoundly impacted by the COVID-19 pandemic and the resulting school closures [2]. School closures generate additional childcare responsibilities for parents who are unable to rely on the external sources of support that school attendance provides. Though children becoming worse during school closures was reported by parents, parents are relevant [16] and accurate [17] sources in reporting their perception on students’ literacy motivation [18].

A questionnaire on demographics was also included regarding age, gender, and SES of the parents, as well as number of children in the family and their school level. Parents’ scores SES were based on school districts of the children, which were calculated by the amount of disposable income, employment opportunities, the ratio of low-income families, and ratio of people who worked in industry and commerce, in the area by the National Developmental Council of Taiwan (2020) [19]. An income of < 800,000 NTD (28,500 USD) per year and an SES score ≤ 40 was considered low; an income of > 800,000 NTD per year and an SES score > 40 was considered high [19].

### Setting and population

The Omicron variant of COVID-19 (SARS-CoV-2) began spreading rapidly in Taiwan following the four-day holiday, Tomb Sweeping Day, in April and in-person classes at 412 schools in 18 cities and counties were cancelled. An online survey was conducted to determine the parent’s perceptions of the impact of these class cancellations on their children and themselves. Data were collected between May 4 and May 9, 2022. Parents were invited to participate in the online survey provided by regional representatives of NAPO. Each parent elects to become a member of the organization by paying an annual fee and providing their email address for as a contact. Because there is a fee to join, typically only one parent in a family is a member. However, both parents could fill out the survey if they shared the link between one another and had separate email accounts. The survey was not distributed by mail, therefore only parents with internet access participated.

### Ethical considerations

The study was approved and granted an exemption from requiring informed consent by the Ethics Committee of Taipei Hospital, Ministry of Health and Welfare (TH-IRB-0021–0001). All methods were performed in accordance with the 1964 Helsinki Declaration and its later amendments or comparable ethical standards.

### Data collection

Heads of the local district organizations throughout Taiwan were notified of the IPRSCCC through the social media site of the NAPO. The district heads then notified their parent members of the survey and provided a link to the cite. Each parent could respond only once, which was ensured by a member of the NAPO, who monitored email accounts from the google form, which was used for the survey. However, the survey data were collected anonymously, thus who actually completed the survey was unknown.

To examine if academic performance had been impaired by school closures and class cancellations, we compared scores from national examinations in Taiwan before and after school closures/class cancellations and compared them with scores from the three previous years, which was inspired by Engzell et al. to examine the impact of school closures on students in the Netherlands [20]. We used two national examinations: the Comprehensive Assessment Program for Junior High School Students (CAP) and the General Scholastic Ability Test (GSAT). The CAP, which is considered a standardized test for 9th grade students, takes place every May and is administered by the MOE. For CAP, there were A, B and C grades. Grade A means proficient, grade B means having basic abilities, and grade C means a need for improvement. We set a cutoff point at grade C. The GSAT takes place every January to test 12th grade students’ knowledge and skills about subjects required students to advance from high school to college. We set a cutoff point at level 6th (A total of 15 levels). Levels 6th means might not have basic knowledge (e.g. score of English was about 40 points, score of Mathematics was about 40 points, and score of Chinese was about 30 points). These measurement methods would be tested for consistency every year, and two data sets are analyzed and posted annually on the Research Center for Psychological and Educational Testing for CAP, and College Entrance Examination Center for GSAT.

### Data analysis

Quantitative data were analyzed by using SPSS version 22.0 for Windows (Armonk, NY: IBM Corp). The null hypothesis for the survey data was there would be no stress difference of school closures/class cancellations on children and parents. Descriptive statistics were used numbers and percentage for demographic data; mean, standard deviations (SD) were used for total scores and item scores on the IPRSCCC. We assumed the survey data from this large sample was continuous. The mean score differences were analyzed with independent t tests. For analysis of continuous and independent variables for more than two groups, data were analyzed with analysis of variance (ANOVA). ANOVA was used to examine the impact of the number of children in the family and scores on the IPRSCCC using Scheffe’s post-hoc test. Significance was set at *p* < 0.05 for statistical comparisons [21].

Qualitative information garnered from the open-ended responses was meticulously gathered and subjected to content analysis. Initially, a pair of researchers independently immersed themselves in the responses, perusing them repeatedly to develop a comprehensive understanding. Subsequently, they extracted preliminary codes directly from the data. Following this, they embarked on a collaborative journey to identify overarching themes. The process culminated in a series of discussions between the two researchers, during which they meticulously compared and refined the emergent themes, working diligently until they reached a mutual agreement.

## Results

### Demographics of responders

A total of 2126 completed IPRSCCC questionnaires were received, which represented 2952 children for an average of 1.73 children/family (SD = 1.02). The age of respondents ranged from ≤ 24 years to > 65 years with most (55%) ranging in age from 36 to 45 years. Most respondents were mothers (*n* = 1704, 80.2%) and most of the children were in kindergarten (aged 4–6 years old, n = 708, 24.0%) or elementary school (aged 7–12 years old, *n* = 1374, 46.5%). When parents were asked about their child’s educational situation, less than half (*n* = 891, 45.8%) reported their child/children had experienced class cancellation. A total of 304 respondents (14.3%) lived in areas with low SES scores. In Taiwan, 85–90% of individuals over the age of 16 years have access to mobile phones and the Internet [22]. Residents in areas with low SES scores represent approximately 10.7% of the total population of Taiwan, and parents from areas with low SES scores comprised 14.3% of the total survey respondents, suggesting these respondents were representative of the Taiwanese population. Demographic details for parents and children are shown in Table 1.

**Table 1:** Demographic data provided by all parents responding to the Impact of the Pandemic-related School Closures/Class Cancellations Survey (IPRSCCC) survey in 2022 (*N* = 2126)

Variable	n	%	Mean	SD
Gender				
Female	1704	80.2%		
Male	422	19.8%		
Age (years)				
≤ 24	43	2.0%		
25–35	268	12.6%		
36–45	1170	55.0%		
46–55	567	26.7%		
56–65	66	3.1%		
> 65	12	0.6%		
Child’s school level				
Kindergarten	708	24.0%		
Elementary school	1374	46.5%		
Junior high school	412	14.0%		
Senior high school	260	8.8%		
University	198	6.7%		
Children per family			1.73	1.02
Child’s educational situation				
Class cancellations	891	45.8%		
In-person classes	1053	54.2%		
Socioeconomic score^a^				
≤ 40	304	14.3%		
> 40	1822	85.7%		

*SD* Standard deviation

^a^Based on the score for the school district in which the parent resided

### Differences in IPRSCCC scores between parents with children whose classes were cancelled and children with in-person classes

To determine the perceived impact of class cancellations on parents and their children, we compared scores on the IPRSCCC between these two groups. The total mean score for impact on children and the item scores were all significantly higher for parents with children whose classes had been cancelled compared with those whose children remained in class (t = 3.590 to 6.337, all *p* < 0.01). Mean total scores for impact on parents were also significantly greater for those whose children’s classes were cancelled compared with those whose children remained in class (t = 2.515, *p* < 0.05). Item scores for emotional stress and no time to rest (t = 2.057, *p* < 0.05 and t = 2.648, *p* < 0.01, respectively) and the total IPRSCCC score (t = 4.646, *p* < 0.01) were also significantly greater for parents with children whose classes were cancelled. Details are shown in Table 2.

**Table 2:** Mean scores for on the Impact of the Pandemic-related School Closures/Class Cancellations Survey (IPRSCCC) for parents with children whose classes were cancelled and those with in-person classes in 2022 (*N* = 2126)

	Cancelled classes	In-person classes		
	(*n* = 891)	(*n* = 1053		
Variable	Mean (SD)	Mean (SD)	t	
**Impact on children (total score)**	19.08 (7.78)	16.98 (7.86)	5.892	** < 0.001**
Item scores				
Disrupted daily routine	6.68 (2.93)	5.90 (2.96)	5.821	** < 0.001**
Learning loss	5.89 (2.83)	5.06 (2.89)	6.337	** < 0.001**
Academic motivation	6.50 (2.97)	6.01 (3.04)	3.590	** < 0.001**
**Impact on parents (total score)**	16.94 (8.00)	16.04 (7.81)	2.515	**.012**
Item scores				
Parent–child conflict	4.65 (2.88)	4.40 (2.84)	1.948	.052
Emotional stress	6.09 (3.05)	5.81 (3.09)	2.057	**.040**
No time for rest	6.20 (3.05)	5.83 (2.98)	2.648	**.008**
**Total IPRSCCC score**	36.02 (14.21)	33.02 (14.18)	4.646	**.001**

*SD* Standard deviation

### Differences in IPRSCCC scores between mothers and fathers with class-cancelled children

We examined if scores on the IPRSCCC differed between fathers (*n* = 176) and mothers (*n* = 621) with class-cancelled children (Table 3). Although scores for perceived impact on children was higher for fathers compared with mothers, only the item score for disrupted daily routine was significantly higher (t = 2.030, *p* < 0.05). No other scores differed significantly.

**Table 3:** Differences in mean scores on the Impact of the Pandemic-related School Closures/Class Cancellations Survey (IPRSCCC) in 2022 between fathers and mothers of children whose classes were cancelled

	Fathers	Mothers		
	(*n* = 176)	(*n* = 621)		
Variable	Mean (SD)	Mean (SD)	t	*p*
**Impact on children (total score)**	20.03 (7.39)	18.76 (7.87)	1.917	0.056
Item scores				
Disrupted daily routine	7.02 (2.84)	6.51 (2.96)	2.030	**0.043**
Learning loss	6.22 (2.69)	5.78 (2.86)	1.816	0.070
Academic motivation	6.79 (2.83)	6.67 (3.00)	1.282	0.200
**Impact on parents (total score)**	17.30 (7.60)	16.85 (8.07)	0.652	0.515
Items scores				
Parent–child conflict	4.68 (2.86)	4.61 (2.89)	0.297	0.767
Emotional stress	6.38 (2.93)	6.00 (3.09)	1.442	0.150
No time for rest	6.23 (2.90)	6.24 (3.05)	-0.021	0.983
**Total IPRSCCC score**	37.32 (13.64)	35.61 (14.28)	1.420	0.156

*SD* Standard deviation

### IPRSCCC scores and number of children per family

We extracted data for parents with children whose classes were cancelled under 18 years of age to examine if there were differences in the impact of the cancellations and the number of children in the family (Table 4). Most parents had one child (*n* = 239, 27.3%) or two children (*n* = 477, 54.4%). Only the item score for impact on emotional stress was significantly greater for parents with two or ≥ 3 children compared with parents with only one child (*F* = 4.225, *p* < 0.05). There were no significant differences for any of the other scores on the IPRSCCC.

**Table 4:** Differences in mean scores on the Impact of the Pandemic-related School Closures/Class Cancellations Survey (IPRSCCC) in 2022 for parents (*N* = 876) with children whose classes were cancelled under the age of 18 years

	Number of children^a^	Scale score			Sheffe’s
Variable		Mean (SD)	F	*p*	Post-hoc test
**Impact on children (Total score)**			1.821	.163	
	1	18.25 (7.780			
	2	19.25 (7.94)			
	≥ 3	19.59 (7.23)			
Item scores					
Disrupted daily routine			1.534	.216	
	1	6.40 (2.93)			
	2	6.74 (3.02)			
	3	6.88 (2.69)			
Learning loss			0.999	.369	
	1	5.66 (2.89)			
	2	5.94 (2.84)			
	3	6.00 (2.74)			
Academic motivation			1.856	.157	
	1	6.19 (3.07)			
	2	6.57 (2.98)			
	3	6.71 (2.82)			
**Impact on parents (total score)**			2.882	0.57	
	1	15.85 (8.27)			
	2	17.30 (7.78)			
	≥ 3	17.30 (8.13)			
Item scores					
Parent–child conflict			1.250	.287	
	1	4.39 (2.89)			
	2	4.74 (2.90)			
	3	4.73 (2.83)			
Emotional stress			**4.225**	**0.015**	**3 = 2 > 1**
	1	5.59 (3.15)			
	2	6.27 (2.97)			
	3	6.24 (3.11)			
No time for rest			1.792	.167	
	1	5.87 (3.17)			
	2	6.30 (2.98)			
	3	6.33 (3.06)			
**Total IPRSCCC score**			2.833	.059	
	1	34.09 (14.43)			
	2	36.55 (14.28)			
	≥ 3	36.89 (13.45)			

*SD* Standard deviation

^a^Number of children: 1 child, *n* = 239; 2 children,* n* = 477; ≥ 3 children, *n* = 160

### IPRSCCC scores and socioeconomic status of parents with class-cancelled children

We examined differences in scores between parents whose children’s classes were cancelled living in areas with low and high SES scores (Supplementary Table S3). Although the score for impact on parents and total IPRSCCC score were higher for parents living in areas with low SES scores compared with parents living in areas with high SES scores, there was no statistical significance between these two groups.

### Personal feedback from parents

Qualitative descriptive findings allow researchers to gain an understanding of the experiences of participants closer to the truth [23]. Content analysis of the open-ended question about class cancellation indicate two categories described concern of the respondents: concerns about COVID-19 vaccines, which was due to the desire for more transparency; and the chaos they experienced from class cancellations, which resulted from a lack of unified and rational standards for cancellations.

Many parents worried the information from the media only conveyed the benefits of vaccination and minimized the risks. Some parents believed the media was passing judgement on parents who were reluctant to vaccinate their children. One father from Lienchiang County (SES score > 40) wrote, “*You still have a chance of being infected by COVID-19 with three doses of the vaccine. The government and the media control the information, and the government should not say that not receiving vaccines is immoral.*” A teacher living in Yilan County (SES score > 40) complained*, “I hope the media could reveal more information about vaccines, instead of only mentioned the benefits of vaccination and the risks without vaccination.”*

Comments about the chaos of class cancellations concerned the inability to make long-term plans. A mother from Taitung County (SES score ≤ 40) wrote, *“The current rolling plans for class cancellations vary from day to day, and there is no unified standard for each county and city. These policies confused me.”* A mother from New Taipei (SES score > 40) wrote the following:

*The policy that students who sat near another student with a confirmed case of COVID within the vicinity of a 3X3 area must leave the classroom, while students outside of the 3X3 vicinity can still go to school, is not proper. Students don’t sit still in the classroom; they move around.*

### The impact class cancellations on academic performance

We examined scores for 9th and 12th grade students on Taiwan’s national examinations in English, Mathematics, and Chinese from 2018 to 2022 to determine if there was an objective impact on academic performance due to class cancellations in 2022 and the previous school closures that had occurred in 2021, as a measure of learning loss. Supplemental Figure S1A shows the percent of students who averaged a Grade of C, which indicates a need for improvement. More 9th grade students needed improvement in 2018 and 2019, than the subsequent three years, suggesting the impact of school closures/class cancellations on learning loss may have not been significant for junior high school students. By contrast, when scores were examined for 12th grade students (Supplemental Figure S1B) in 2021 and 2022, the percent of students needing improvement (levels ≤ 6) on the English section of the GSAT was greater than 2018 to 2020; and the percent of students needing improvement in mathematics (levels ≤ 6) was also greater in 2021. It is not clear if there was a substantial change in performance on the Chinese section of the exam. The impact of school closures/class cancellations on learning loss seemed to be greater for senior high school students than junior high school students.

## Discussion

**Fig. 2:**
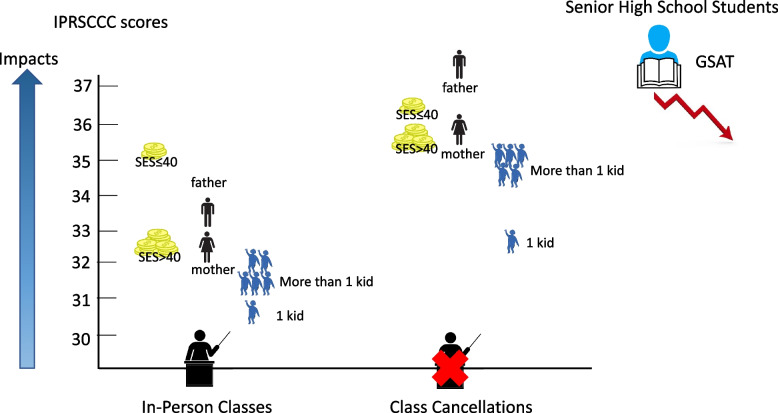
Total scores on the IPRSCCC were significantly higher for parents having children whose classes had been cancelled compared with parents having children with in-person classes. More 12^th^ grade senior high school students received scores ≤ 6 scores in English, mathematics and Chinese subjects on the General Scholastic Ability Test (GSAT)

Longitudinal studies designed to realize the social and mental stress of the emerging COVID-19 crisis can provide timely information to governments and communities [24]. The scores from the online IPRSCCC survey provided the Taiwanese government and the public with information on the impact of class cancellations in April 2022 (Fig. 2). When compared with parents whose children were allowed to continue with in-person classes, IPRSCCC scores indicated parents perceived the class cancellations of the child/children disrupted daily routine, learning loss and impacted academic motivation. They also reported emotional stress and no time for rest, which were associated with parental burnout [25, 26]. When scores for mothers and fathers of children whose classes were cancelled, disrupted daily routine was significantly greater for fathers. Our findings also indicated that the impact of class cancellations was greater for the item score of emotional stress for parents with two or three children compared with parents with parents with only one child. Finally, national exam scores indicated there was learning loss for students in senior high school but not junior high school (Fig. 2).

Parents perceptions are considered to be relevant [16] and accurate [17] sources for a child’s academic motivation [18]. The COVID-19 pandemic and the associated restrictive measures (i.e., home confinement, school closures, and distance learning) may have further hindered students’ ability to sustain academic motivation towards school activities, such as attending online and asynchronous classes, studying, and doing homework [27]. Although the impact on parents and children was not as significant as we expected, the cancellations were relatively short-term, especially when compared with the nationwide school closures that occurred in Taiwan from May to July 2021 [4]. However, children in remote and low-income areas suffered from lack of computers and poor Internet service, which significantly influenced remote learning, which resulted from “dropping out online”. According to the data from the MOE, one of every six students failed to attend online classes during the nationwide school closures that occurred in 2021 [28]. Therefore, there is the potential for learning loss of children in remote and low-income areas, which should be a concern for the Government.

One other explanation for the lower impact of class cancellations on parents in April of 2022 may be the previous experience of the school closures due to the alpha variant of COVID-19 in the second half of 2021. Parents may have become better at adapting to the changes in the family dynamics that were required during the long-term nationwide school closures and strict quarantine in 2021 [4]. However, the pandemic still impacted the lives (child/children disrupted daily routine, learning loss, impacted academic motivation, emotional stress and no time for rest) of parents with children whose classes had been cancelled much more, compared with parents whose children remained in class. Our study extended the current understanding of how the pandemic, specifically class cancellation (school closures), has impacted the lives of parents and their children.

The mobility restriction and social isolation associated with quarantine are major concerns for families' psychological wellbeing. Parents reported a high presence of psychological distress such as depression, stress, irritability, and post-traumatic stress symptoms associated with quarantine [29]. An Italian study found that parents who found managing life under quarantine conditions difficult reported feeling stressed [30]. More than 50% of parents reported being stressed by social distancing and the closure of schools and childcare facilities in Germany [31]. A global survey of 114 parents from 31 countries found quarantine due to COVID-19 resulted in parental stress and symptoms of anxiety in their children, which included focus reduction, sleeping difficulties, and appetite changes [32]. Therefore, Taiwan’s effort to coexist with the virus by enacting fewer social restrictions and measures may have lessened the pressure on parents during class cancellations in 2022.

Parents with two, three and more children perceived a greater emotional stress in our study. The presence of children in the household was associated with higher levels of stress and anxiety for mothers during COVID-19 [33]. Parental stress increased significantly during the pandemic [31]. Subgroups of parents also reported very high levels of depressive symptoms (12.3%) and anxiety (9.7%) [31]. Mothers of three children have greater levels of stress compared with mothers of one or two children [34].

The open-ended question in the survey resulted in comments from parents that focused on vaccines and standards for class cancellations. The parents urged the government and the media to give provide a more complete and transparent information about the vaccines targeting the novel corona virus. Parents wanted to know about the benefits and adverse effects of vaccines. Parents worried that plans for rolling class cancellations would affect their children’s health and learning and asked for more unified standards. They also criticized the mandate of “a 3X3 area” for exposure as somewhat arbitrary. In May of 2022, parent organizations called on the government to unify the criteria of class cancellations and online-teaching [35]. Following these requests, the MOE cancelled “prevention leave of the vicinity of a 3X3 area” in schools [36], and the criteria of class cancellation of each local governments were posted on the website [36].

Cancellation of face-to-face class instruction during the COVID-19 pandemic has led to concerns about consequences for students' learning [20]. Data from Australia revealed learning loss of about 3 percentile points [24]. A negative effect of school closures on student achievement, specifically in younger students and students from families with low socioeconomic status was found [11]. Our data demonstrated that the impact of school closures/class cancellations on learning from 2021 to 2022 was greatest for senior high school students. However, junior high school students seemed to adapt well. Similar findings were seen in The Program for International Student Assessment (PISA). Taiwan’s PISA results, about 6,000 15-year-old students (grade 9th) participating in the test, were above average for the Organization for Economic Cooperation & Development (OECD). It correlated to our finding that these junior high school students successfully adapted to the realities of education during COVID [37]. This may be due to that the nationwide school closures or class cancellations were relatively short-term in Taiwan compared with other countries [4].

Though we Taiwan didn’t have national examination for the elementary school students, 70% of parents believe that their children's academic performance has declined during the class cancellations; more than 30% of children are dissatisfied with remote-learning [38]. These were self-reported information and not easily comparable to the standardized achievement data. Whether learning loss is even greater for children in the elementary schools, and low-income areas with weaker infrastructure in Taiwan will require further study.

The COVID-19 pandemic has impacted parents worldwide and has had negative effects on children’s physical and mental health [2, 7]. Parents of children receiving virtual or combined instruction more frequently reported that their child’s mental or emotional health worsened during the pandemic [9], especially in high school [10]. This survey confirmed the negative impact of class cancellations in Taiwan, which resulted from the surge in cases of the Omicron variant of COVID-19 in April 2022. Parents’ opinions are considered to be important for policy-making in Taiwan. NAPO previously warned of the danger of children under 12 years old staying at home alone during school closures, therefore, the local government prohibited preventive school closures/class cancellations in elementary schools in 2022.

### Limitations

The findings of this study have some limitations. First, the self-report survey data was analyzed without direct quantitative measures of the students’ learning loss. Although we examined scores from Taiwan’s national examinations for 9th and 12th grade students, these data do not provide support for parents’ subjective assessments. In addition, there are no national examinations for elementary school children. Collecting test score information for each child in future studies could confirm the degree of learning loss. Second, our survey was written in Chinese, which does not cover the linguistic diversity of immigrants living in Taiwan. Third, the feelings, worries, and needs of the parents’ children were assessed indirectly through the parents’ subjective perceptions. Child self-report would also be valuable for understanding how they were impacted by the pandemic, especially important for internalizing states and in relation to school-specific experiences. Fourth, online surveys cannot accurately describe the population to which the surveys are distributed, and the respondents may be biased [39]. Fifth, the SES of school district may not correlate with the household income. Sixth, obtaining adaptation results by age group/developmental stage was not possible due to confounding variables introduced by children in different educational levels within the same families.

## Conclusion

Significantly higher mean IPRSCCC scores for parents of children whose classes were cancelled adds additional evidence of the impact of class cancellations during the COVID-19 pandemic. Learning loss for senior high school student due to school closures and class cancellations was demonstrated by the greater percentage of students with scores on national exams indicated a need for improvement, however, the impacts of the learning loss seemed to be less in the junior high school students. Further evaluations are warranted. Analysis of academic test scores to investigate learning loss during school closures or class cancellations, and closely monitoring student progression should continue in the years to come [40]. In addition, the government of Taiwan should encourage schools to assess whether in-person courses are necessary and gauge the effectiveness of remote learning [41] while finding ways to accommodate those who lack Internet access.

### Supplementary Information

Supplementary Material 1: Figure S1. Scores for English, Chinese, and Mathematics from 2018 to 2022. (A) The percent of 9th grade students with grade C on the Comprehensive Assessment Program (CAP), indicating a need for improvement. Source: The Research Center for Psychological and Educational Testing. (B) The percent of 12th grade students with scores ≤ 6 (maximum score = 15) on the General Scholastic Ability Test (GSAT). Source: College entrance examination center. *Mathematics tests are separated into test A (difficult) and test B (easy). We chose test B (easy) for comparisons.

Supplementary Material 2.

## Data Availability

The datasets generated and/or analyzed during the current study are available from the corresponding author on reasonable request.
