# Supplementary material for: Impact of class cancellations on parents’ and children’ adaptation following an outbreak of the Omicron variant during the COVID-19 pandemic in Taiwan in April 2022
Source: BMC Public Health. 2024 Jul 16;24:1902. doi: 10.1186/s12889-024-18976-y (PMC11251347; doi:10.1186/s12889-024-18976-y)
Supplement: Supplementary file 1 — Supplementary Material 1: Figure S1. Scores for English, Chinese, and Mathematics from 2018 to 2022. (A) The percent of 9th grade students with grade C on the Comprehensive Assessment Program (CAP), indicating a need for improvement. Source: The Research Center for Psychological and Educational Testing. (B) The percent of 12th grade students with scores ≤ 6 (maximum score = 15) on the General Scholastic Ability Test (GSAT). Source: College entrance examination center. *Mathematics tests are separated into test A (difficult) and test B (easy). We chose test B (easy) for comparisons. [file 12889_2024_18976_MOESM1_ESM.pptx]

## Slide 1
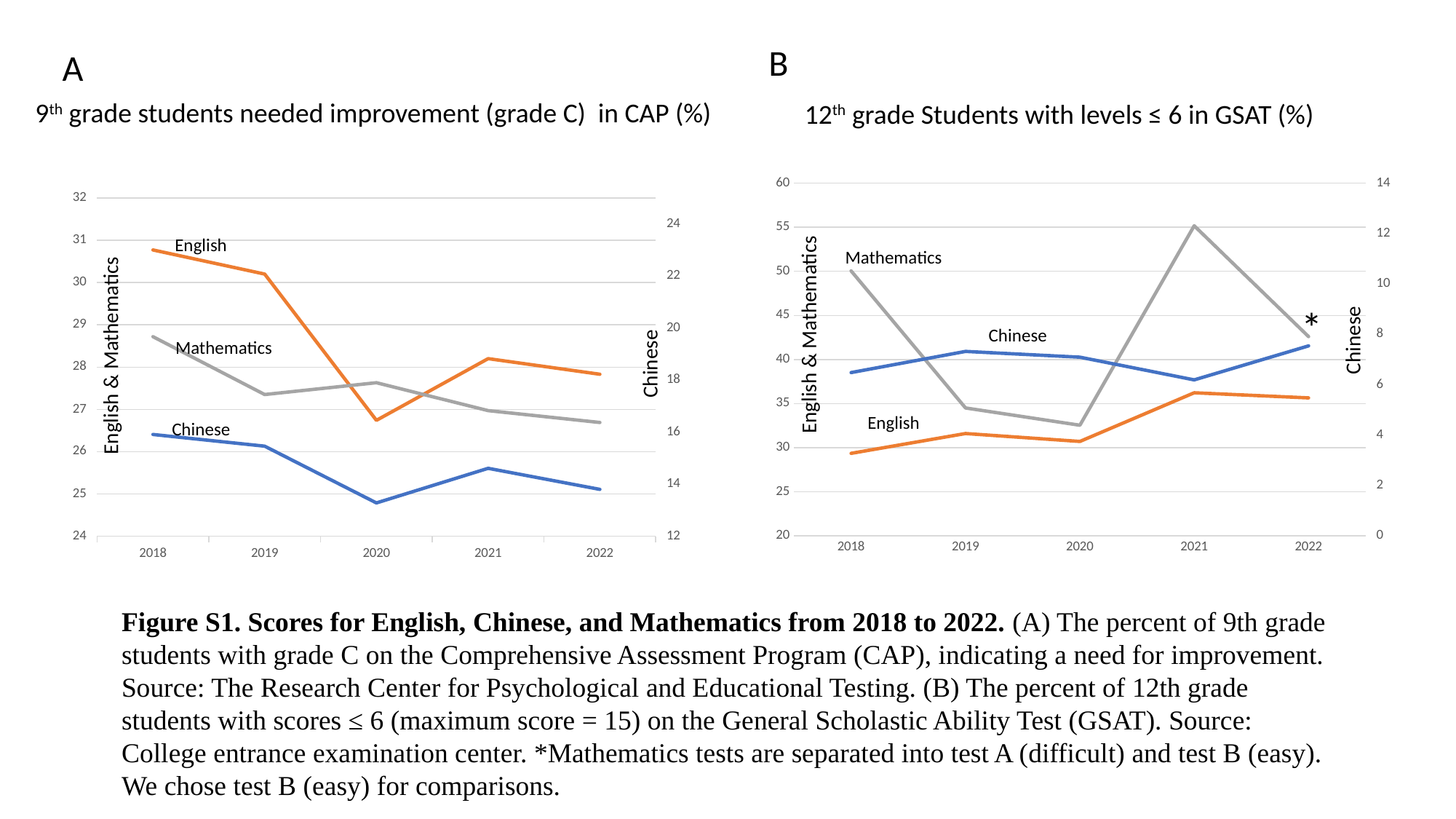

B
A
### Chart
| Category | English | Mathematics | Chinese |
|---|---|---|---|
| 2018 | 29.349999999999998 | 50.05 | 6.4799999999999995 |
| 2019 | 31.600000000000005 | 34.5 | 7.319999999999999 |
| 2020 | 30.700000000000003 | 32.53999999999999 | 7.089999999999999 |
| 2021 | 36.22 | 55.17 | 6.1899999999999995 |
| 2022 | 35.63999999999999 | 42.59 | 7.54 |9th grade students needed improvement (grade C) in CAP (%)
12th grade Students with levels ≤ 6 in GSAT (%)
### Chart
| Category | English | Mathematics | Chinese |
|---|---|---|---|
| 2018 | 30.77 | 28.72 | 15.91 |
| 2019 | 30.2 | 27.35 | 15.46 |
| 2020 | 26.74 | 27.63 | 13.28 |
| 2021 | 28.2 | 26.97 | 14.61 |
| 2022 | 27.83 | 26.69 | 13.8 |English
Mathematics
*
English & Mathematics
Chinese
Chinese
Mathematics
English & Mathematics
Chinese
English
Chinese
Figure S1. Scores for English, Chinese, and Mathematics from 2018 to 2022. (A) The percent of 9th grade students with grade C on the Comprehensive Assessment Program (CAP), indicating a need for improvement. Source: The Research Center for Psychological and Educational Testing. (B) The percent of 12th grade students with scores ≤ 6 (maximum score = 15) on the General Scholastic Ability Test (GSAT). Source: College entrance examination center. *Mathematics tests are separated into test A (difficult) and test B (easy). We chose test B (easy) for comparisons.
