# Supplementary material for: Impact of class cancellations on parents’ and children’ adaptation following an outbreak of the Omicron variant during the COVID-19 pandemic in Taiwan in April 2022
Source: BMC Public Health. 2024 Jul 16;24:1902. doi: 10.1186/s12889-024-18976-y (PMC11251347; doi:10.1186/s12889-024-18976-y)
Supplement: Supplementary file 2 — Supplementary Material 2. [file 12889_2024_18976_MOESM2_ESM.docx]

**Table S1** Factor loadings for the Pandemic-related School Closures/Class Cancellations Survey (IPRSCCC)

| IPRSCCC item | Factor loadings |
| --- | --- |
| **Impact on children** |  |
| 1.Disrupted daily routine | 0.732 |
| 2.Learning loss | 0.850 |
| 3.Academic motivation | 0.887 |
| **Impact on parents** |  |
| 4.Parent-child conflict | 0.688 |
| 5.Emotional stress | 0.867 |
| 6.No time for rest | 0.697 |

**Table S2** The overall fit indices of IPRSCC

|  | �2 X^2^ (df) | GFI | AGFI | NFI | CFI | SRMR | TLI | RMSEA |
| --- | --- | --- | --- | --- | --- | --- | --- | --- |
| IPRSCCC | 30.638(8) ** | 0.985 | 0.961 | 0.984 | 0.952 | 0.034 | 0.977 | 0.084 |

GFI, goodness-of-fit index; AGFI, adjusted GFI; NFI, normed fit index; CFI, Comparative Fit Index: SRMR, standardized root-mean-square residual; TLI, Tucker Lewis Index: RMSEA, root mean square error of approximation.

** means *p* < 0.01.

**Table S3** Differences between mean scores on the Impact of the Pandemic-related School Closures/Class Cancellations Survey (IPRSCCC) in 2022 for parents with children whose classes were cancelled living in areas with SES scores above and below 40

|  | SES score | |  |  |
| --- | --- | --- | --- | --- |
|  | > 40 | ≤ 40 |  |  |
|  | (n = 800) | (n = 91) |  |  |
| Variable | Mean (SD) | Mean (SD) | t | *p* |
| **Impact on children (total score)** | 19.19 (7.84) | 19.07 ( 7.15) | 1.307 | .192 |
| Item scores |  |  |  |  |
| Disrupted daily routine | 6.75 (2.94) | 6.12 (2.80) | 1.928 | .054 |
| Learning loss | 5.94 (2.86) | 5.42 (2.60) | 1.680 | .093 |
| Academic motivation | 6.50 (2.98) | 6.53 (2.89) | -0.880 | .936 |
| **Impact on parents (total score)** | 16.78 (8.03) | 18.35 (7.60) | -1.774 | .076 |
| Item scores |  |  |  |  |
| Parent-child conflict | 4.61 (2.89) | 5.00 (2.85) | -1.211 | .226 |
| Emotional stress | 6.04 (3.05) | 6.57 (3.04) | -1.578 | .115 |
| No time for rest | 6.13 (3.07) | 6.78 (2.81) | -1.928 | .540 |
| **Total IPRSCCS score** | 35.97 (14.35) | 36.42 (12.99) | -0.282 | .778 |

*Note:* SD = standard deviation
